# The Protective Effects of Peroxisome Proliferator-Activated Receptor Gamma in Cerebral Ischemia-Reperfusion Injury

**DOI:** 10.3389/fneur.2020.588516

**Published:** 2020-11-17

**Authors:** Yanping Ding, Jie Kang, Shuning Liu, Yuqin Xu, Baoping Shao

**Affiliations:** ^1^College of Life Science, Northwest Normal University, Lanzhou, China; ^2^College of Life Science, Lanzhou University, Lanzhou, China

**Keywords:** cerebral ischemia-reperfusion, peroxisome proliferator-activated receptor γ, anti-inflammation, anti-oxidative stress, microglia activation, anti-apoptosis

## Abstract

Cerebral ischemia-reperfusion injury (CI/RI) is a complex pathological process that often occurs secondary to trauma, surgery, and shock. Peroxisome proliferator activated receptor gamma (PPARγ) is a subunit of the PPAR and is a ligand-activated nuclear transcription factor. After being activated by its ligand, PPARγ can combine with specific DNA response elements to regulate the transcription and expression of genes. It has a wide range of biological functions, such as regulating lipid metabolism, improving insulin sensitivity, modulating anti-tumor mechanisms, and inhibiting inflammation. In recent years, some studies have shown that PPARγ exerts a protective effect during CI/RI. This article aims to summarize the research progress of studies that have investigated the protective effects of PPARγ in CI/RI and the cellular and molecular mechanisms through which these effects are modulated, including inhibition of excitatory amino acid toxicity, reduced Ca^2+^ overload, anti-oxidative stress, anti-inflammation, inhibition of microglial activation, maintain the BBB, promotion of angiogenesis, and neurogenesis and anti-apoptotic processes.

## Introduction

Cerebral ischemia-reperfusion injury (CI/RI) is a pathological process characterized by an initial restriction of blood supply to the brain, followed by the subsequent restoration of perfusion along with concomitant reoxygenation, resulting in severe damage to brain tissue (1). CI/RI is a very common pathophysiological phenomenon in the clinic, and is commonly observed in patients with traumatic injuries or in association with certain disease processes; some examples include hemorrhagic shock, severe surgical trauma, tumor resection, and tissue/organ transplantation (2). CI/RI is associated with certain characteristics that include high degrees of morbidity, disability, and mortality, and a high recurrence rate, which exerts a heavy burden on the patient's family and society as a whole (3). At present, the cellular and molecular mechanisms through which CI/RI occurs have not been fully elucidated, but CI/RI is believed to be related to the production of oxidative free radicals, mitochondrial damage, disorders of energy metabolism, Ca^2+^ overload, and excitotoxicity (4). These pathological processes aggravate neuroinflammation and promote the activation of microglia, which is followed by the production of multiple pro-inflammatory mediators, such as cytokines, and chemokines involved in inducing changes in blood-brain barrier (BBB) permeability, causing edema, apoptosis, or necrosis.

Peroxisome proliferator-activated receptors (PPARs) are ligand-inducible nuclear transcription factors that belong to the type II nuclear receptor superfamily. PPARs include three heterologous forms, namely PPARα, PPARβ/δ, and PPARγ, with PPARγ being the most intensively studied to date (5). PPARγ is widely distributed throughout the body, mainly in adipose tissue, but it is also expressed in small amounts in the spleen and liver, in monocytes/macrophages, B cells, T cells, and smooth muscle cells (6). In the nervous system, PPARγ is expressed in neurons and glia, including microglia and astrocytes, in multiple brain regions (7). When activated by its ligands, PPARγ can bind to specific DNA-response elements to regulate gene transcription and expression, thereby modulating a variety of physiological functions, including participating in lipid and glucose metabolism, improving insulin sensitivity, and promoting adipocyte differentiation (8).

Recent studies have shown that PPARγ exerts neuroprotective effects in CI/RI by downregulating proinflammatory mediators, modulating selective activation of immune cells, inducing antioxidant expression, participating in the proliferation, and differentiation of neuronal stem cells, increasing the expression of vascular endothelial growth factor (VEGF), maintaining the BBB, and reducing the expression of apoptotic factors (7). At present, PPARs agonists have been used in clinical trials mainly for the treatment of hyperlipidemia, diabetes and metabolic syndrome, and achieved results. A national cohort study showed that Asian patients with ischemic stroke taking pioglitazone for type 2 diabetes mellitus (T2DM) could lower the risks of recurrent ischemic stroke during the long term follow-up. And their data provided the evidence of pioglitazone for secondary prevention of ischemic stroke in Asian T2DM patients, which provides a theoretical basis for the development of new cardiovascular and cerebrovascular disease drugs (9). The research progress of PPARγ agonists in the treatment of CI/RI in the last 5 years is summarized in the Table 1 (10–26).

**Table 1:** Summation of the research progress of PPARγ agonists in the treatment of CI/RI in the last 5 years.

**Ligand**	**Model**	**Treatment time points and dose**	**Molecular**	**Treatment outcomes**	**References**
Pioglitazone (PGZ)	BCCAO for 30 mins + reperfusion for 24 h (rat)	14 days prior MCAO, 10 mg/kg/d, p.o.	Reduced MDA, TNF-α, iNOS, and caspase 3 expression; Increased glutathione (GSH) expression	Anti-inflammatory; Anti-apoptosis; Improved the neurological functions; Reduce morphological damage	(10)
	MCAO for 90 mins and reperfusion for 3 days (rat); OGD for 2 h (primary cultured astrocytes)	3 days after MCAO, 10 mg/kg/d, i.p.; 1 h prior OGD, 10 μM	Suppression of HMGB-1/RAGE and Rac1/ROS Pathway	Anti-inflammatory; Anti-apoptosis; Anti-oxidation; Reduce morphological damage Improved the neurological functions;	(11)
	MCAO for 90 mins + ovariectomized rat	7–14 days after MCAO, 2.5 mg/kg/d, i.p.	Activation of Akt, MAP2, and VEGF	Anti-inflammatory; Promote regeneration	(12)
	MCAO for 90 mins and reperfusion for 24 h (rat)	3 days after reperfusion, 10 mg/kg/d, p.o.	Reduced caspase 1, NLRP3, IL-1β, and IL-18 expression	Anti-inflammatory; Anti-apoptosis	(13)
	MCAO for 2 h and reperfusion for 24 h (rat)	7 days prior MCAO, 15 mg/kg/d, i.p.	Reduced caspase-1, Gasdermin D, IL-1β, and IL-18 expression	Anti-inflammatory; Anti-apoptosis; Reduce morphological damage	(14)
Rosiglitazone (RSG)	MCAO for 2 h + tPA-induced hemorrhagic transformation (2 h after MCAO) (mouse)	1 h prior MCAO, 6 mg/kg, i.p.	Attenuates HT and BBB disruption; Reduced iNOS expression; Increased CD206 expression	Regulate microglial phenotype Reduce morphological damage	(15)
	OGD/R for 10 h reperfusion for 24 h (PC12 cells)	10 h after OGD, 10 μmol	Reduced HMGB1 expression Increased DUSP8 and Bcl-xl expression	Anti-inflammatory; Anti-apoptosis	(16)
	Spontaneousintracerebral hemorrhage (ICH) (rat)	3 days prior MCAO **+** 1 h, 2 days and 3 days after MCAO, 3 mg/Kg/d, p.o.	Increased CD36 expression	Activate phagocytic ability of microglia; Improved the neurological functions	(17)
Propane-2-sulfonic acid octadec-9-enyl-amide (N15)	MCAO for 2 h (rat)	2–13 days after MCAO; 100 mg/kg/d, p.o.	Increased GAP-43, synaptophysin(SYP), BDNF, and NT-3 expression in the hippocampus	Promote regeneration	(18)
N15	MCAO for 90 mins (mouse)	2 h after reperfusion 200 mg/kg, i.p.	Inhibition of the NF-κB, STAT3, and ERK1/2 signaling pathways	Anti-inflammatory; Inhibit microglial activation Reduce morphological damage; Improved the neurological functions;	(19)
Oleic acid (OA)	MCAO for 90 mins (rat); Photothrombosis 15 mins (mouse); Four-vessel occlusion (4-VO) 10 mins (rat)	0, 2, and 3 h after surgery, 10, 30 mg/kg; 20, 60, 200 mg/kg; 1, 30, 10 mg/kg, i.p.	Reduced COX-2, iNOS, and TNF-α expression	Anti-inflammatory; Anti-apoptosis; Promote cognition; Reduce morphological damage	(20)
10-O-(N,N-dimethylaminoethyl)-ginkgolide B methanesulfonate(XQ-1H)	MCAO + reperfusion for 1 or 3 days (mouse); OGD/R for 3 h (BV-2 microglia)	1, 3 days after MCAO, TID, 31, 62 mg/kg, p.o; 24 h before OGD/R, 1, 3, 10 μM	Regulation of microglia polarization	Anti-inflammatory; Promote cognition; Anti-oxidation; Reduce morphological damage; Regulate microglial phenotype	(21)
Telmisartan (TEL)	MCAO for 2 h (rat)	3 weeks after MCAO, 1, 5, 10 mg/kg/d	Reduced MMP2, MMP9, and acetylcholinesterase (AChE) expression; Increased choline acetyltransferase (ChAT) and SYN expression	Promote regeneration; Promote cognition	(22)
Ginsenoside Rg1	MCAO for 90 mins and reperfusion for 24 h (rat); OGD for 90 mins reperfusion for 24 h (neurons)	6 h after reperfusion, 30, 60 mg/kg i.p.; 30, 60 μmol/L	Reduced TNF-α, IL-6,NF-κB, and MPO expression; Increased antioxidant enzymes SOD and CAT expression	Anti-inflammatory; Anti-oxidation Reduce morphological damage	(23)
15-hydroxyeicosatetraenoic acid (15-HETE)	MCAO for 1 h and reperfusion for 24 h (rat)	30 mins prior MCAO, 15 μl, icv.	Reduced MDA expression; Increased SOD expression	Anti-apoptosis; Anti-oxidation; Reduce morphological damage	(24)
Liraglutide	MCAO with diabetes mellitus (rat)	7 days prior MCAO, BID, 100 μg/kg, i.p.	Reduced TNF-α and NF-κB expression	Anti-inflammatory; Decreased glucose	(25)
15d-PGJ2	MCAO with diabetes mellitus (rat)	21 days prior MCAO + 3 h, 6 days after MCAO, 200 μg/kg/d, i.p.	Reduced of CD68, TNF-α, and IL-1β expression	Anti-inflammatory; Anti-apoptosis; Inhibit microglial activation	(26)

In this review, we summarize major progress made to date toward understanding the neuroprotective mechanism exerted by PPARγ in CI/RI in terms of its involvement in reducing glutamate toxicity and Ca^2+^ overload, anti-oxidative stress, anti-inflammation, including its role in inhibiting microglial activation, maintaining the BBB, promoting angiogenesis and neurogenesis, and anti-apoptotic processes.

## The Role of PPARγ in Reducing Glutamate-Induced Toxicity and Ca^2+^ Overload During CI/RI

Glutamate (Glu) is one of the most widely distributed excitatory amino acids (EAAs) in the central nervous system (CNS) (4), and it plays an important role in the development and maturation of various cells under normal physiological conditions. However, excessive levels of glutamate in the intercellular space after cerebral ischemia-hypoxia leads to overexcitation of glutamate receptors, which mediate the toxic effects of EAAs, including neuronal death and oligodendrocyte damage (4, 27). Following acute and chronic CNS insults, N-methyl-D-aspartic acid (NMDA) receptor-mediated neurotoxicity results in the production of oxygen free radicals and NO (28). These effects lead to decreased membrane permeability, increased Ca^2+^ influx, calcium overload, reactive oxygen species (ROS) aggregation, mitochondrial injury, increased BBB permeability, and cytotoxic brain edema, all of which result in the induction of cellular necrosis and apoptosis (4). The mechanism for maintaining extracellular glutamate concentrations below excitotoxic levels in the CNS is the upregulation of a certain glutamate transporter protein expressed on astrocytes, known as excitatory amino acid transporter 2 (EAAT2), which is responsible for clearing up to 90% of all extracellular glutamate (27). Therefore, the upregulation of glutamate transporter 1 (GLT1)/EAAT2 and other glutamate transporter proteins after cerebral ischemia and hypoxia may have significant benefits.

Recent studies have shown that PPARγ activation can play important roles in all the processes mentioned above. For example, Romera et al. (29) showed that ischemic preconditioning caused an increase in nuclear PPARγ transcriptional activity in neurons and astrocytes in neuronal-astrocytic co-cultures, and the PPARγ agonist rosiglitazone increased both GLT-1/EAAT2 mRNA and protein expression and glutamate uptake, and reduced oxygen-glucose deprivation (OGD)-induced cell death and glutamate release. In addition, the PPARγ agonist rosiglitazone protects oligodendrocyte precursor cells (OPCs) and oligodendrocytes by regulating the glutamate transporter proteins GLT1/EAAT2 to blunt the extent of excitotoxic injuries (30). According to the research, telmisartan, an angiotensin-receptor blocker (ARB) drug, also upregulates pigment epithelium-derived factor (PEDF) by activating PPARγ after middle cerebral artery occlusion (MCAO), and improves the associated neurological deficits, reduces cerebral edema, promotes the expression of glutamate transporters [both GLT-1 and glutamate-aspartate transporter (GLAST)], and reduces the activation of microglia and the expression of pro-inflammatory factors such as interleukin 1 β (IL-1β), interleukin 6 (IL-6), tumor necrosis factor alpha (TNF-α), cyclooxygenase-2 (COX-2), and inducible nitric oxide synthase (iNOS) (31). Garcia-Bueno et al. (32) reported that treatment with PPARγ agonists exerted a direct protective effect on cerebral glucose and glutamate metabolism, and PPARγ agonists reduced oxidative damage in the brain under stress by increasing the expression of neuronal glucose transporter protein 3 (GLUT-3) and by regulating EAAT-2.

Calcium overload causes CI/RI, and there are several key mechanisms responsible for the changes: (i) increased activity of mitochondrial calcium pumps and excessive intake of Ca^2+^ leads to increased adenosine triphosphate (ATP) consumption, the opening of mitochondrial permeability transition pores, the interference of mitochondrial oxidative phosphorylation, and ultimately mitochondrial dysfunction; (ii) the increased intracellular Ca^2+^ concentration activates a variety of phospholipases, which promote the decomposition of membrane phospholipids, followed by structural damage to the cell and organelle membranes; (iii) the increased intracellular Ca^2+^ promotes the generation of oxygen free radicals by enhancing the activity of Ca^2+^-dependent protein kinase (CaMK), and the interaction between ROS and Ca^2+^ lasts for hours or even days after cerebral ischemia, eventually triggering cell death (33, 34).

Studies have shown that berberine reduced calcium overload caused by cerebral ischemia by mediating PPARγ (35). The PPARγ activator G-Rg1 downregulates the increased free radical concentrations induced by excessive Glu and asparaginic acid (Asp) levels, inhibits the influx of extracellular calcium as well as neuronal NOS (nNOS) activity, and attenuates ischemic nerve damage and its associated apoptotic effects (such as further intracellular calcium overload, alpha-aminoadipic acid (AAA; a glutamate analog) toxicity, energy metabolism disorder, and mitochondrial apoptosis) via its effects on NMDA receptors, endoplasmic reticulum (ER) stress, and the 5' AMP-activated protein kinase (AMPK)-associated AMP/AMPK-GLUT pathways (4).

In summary, PPARγ can reduce Ca^2+^ overload by increasing the expression of EAAT2, reducing the release of glutamate and the activation of NMDA receptors, and attenuates neuronal necrosis and apoptosis after CI/RI.

## The Role of PPARγ in Anti-Oxidative Stress/Anti-ER Stress Responses During CI/RI

Although oxidative stress, inflammation, and apoptosis occur concurrently in ischemic stroke, oxidative stress seems to be a potential initiator (36). ROS are produced in the early stages of CI/RI (37) and promote the expression of multiple pro-inflammatory mediators, including monocyte chemoattractant protein 1 (MCP-1), vascular cell adhesion molecule-1 (VCAM-1), and IL-6 by activating nuclear factor-κB (NF-κB), extracellular signal-regulated protein kinases (ERK1/2), protein tyrosine kinase (PTK), and Janus kinase 2-signal transducers and activators of transcription (JAK2-STAT) (38, 39). In addition, the production of ROS leads to increased leakage of malonaldehyde (MDA) and lactate dehydrogenase (LDH) on intracellular biofilms, and irreversible destruction of proteins and nucleic acids (40).

Activation of nicotinamide adenine dinucleotide phosphate (NADPH) oxidase (NOX), of the non-phagocytic cell oxidase family, is a major driving force in ROS generation in the reoxygenation/reperfusion environment (41). Seven homologous members have been documented in the family: Nox1-5 and Duox1-2; among them, Nox2 was the first member to be identified and has been the most extensively studied. In its resting state, the catalytic subunit of Nox2 remains bound to the regulatory subunit of p22phox [human neutrophil cytochrome b light chain (CYBA)], anchored in the plasma membrane. Upon stimulation, the cytosolic subunits p47phox, p67phox, p40phox, and small Rac GTPases (rac1/2) are recruited to the plasma membrane where they bind to Nox2/p22phox to form the active NOX holoenzyme (41). It has been proven that a sequential neuroprotective signaling cascade activated by the PPARγ agonist 15d-PGJ2 reduces NF-κB nuclear translocation to suppress the NF-κB-driven p22phox transcription and subsequent NOX activation, thereby decreasing the production of ROS (41). Ras-related C3 botulinum toxin substrate l (Rac1), a small GTPase protein, is an essential subunit of NOX responsible for the generation of ROS, with its active form being Rac1-GTP (42). Xia et al. (11) found that the PPARγ agonist pioglitazone inhibited the activity of Rac1, the production of ROS, and the apoptosis induced by ischemia/hypoxia in both the MCAO and *in vitro* OGD models, indicating that PPARγ inhibits the activation of Rac1/ROS signaling.

In addition to reducing the production of superoxide compounds by reducing NOX, PPARγ also accelerates the removal of excess superoxide molecules by increasing the production of antioxidant enzymes. Superoxide dismutase (SOD), glutathione (GSH) peroxidase (GSH-Px), and catalase (CAT) are all endogenous antioxidant enzymes that constitute the first line of the intracellular antioxidant defense system, which can inhibit free radical damage by removing excess ROS (43). The CAT and SOD gene promoters contain peroxisome proliferator response element (PPRE), indicating that they are directly regulated by PPARγ (44, 45). Furthermore, the depletion of GSH was prevented by both pioglitazone and rosiglitazone treatment in adult rats subjected to cerebral ischemia (44, 46). Studies have shown that ginsenoside G-Rg1 might be a potent agonist of PPARγ, with demonstrated sensitivity and protective properties against oxidative stress in oxidative tissue injury due to its significant reduction of myeloperoxidase (MPO) activity and the normalization of the diminished expression of antioxidant enzymes SOD and CAT (23). The PPARγ antagonist GW9662 blocked the increase in PPARγ DNA binding activity and the antioxidant enzymatic activities of SOD and CAT, thereby abolishing the protective effect of PPARγ activation in the OGD model with exposed neurons (47). Shimazu et al. (48) showed that PPARγ activation could increase the activity of Cu-Zn SOD and its free radical scavenging effect, reducing the levels of NO, COX-2, iNOS, and nitrotyrosine in cerebral ischemia models (49). Uncoupling proteins (UCPs) are located in the inner mitochondrial membrane that can prevent mitochondrial damage by reducing the proton gradient and ROS production through uncoupled oxidative phosphorylation. It has been shown that PPARγ is involved in the transcriptional regulation of UCPs (50).

In addition to oxidative stress, ER stress is also crucial to the development of CI/RI. Ischemic conditions can stimulate unfolded protein aggregation in the ER lumen, leading to the unfolded protein response (UPR). The UPR is correlated with ER membrane extension, promoting degeneration of unfolded proteins, inhibiting protein synthesis, and enhanced folding chaperon transcription. Although the ER plays an important role in restoring homeostasis, the UPR can lead to accelerated activation of cell death-promoting pathways regulated by CCAAT/enhancer binding protein (C/EBP) homologous protein (CHOP; a marker of apoptosis in ER-associated stress mechanisms), c-Jun N-terminal kinase (JNK), and caspase-12 under conditions of severe or prolonged stress (51). The stimulated caspase-12 subsequently stimulates caspase-9 as well as caspase-3, triggers fragmentation of DNA, and ultimately causes cell death. CHOP upregulates B-cell lymphoma 2 (Bcl-2)-associated X protein (BAX) while inhibiting Bcl-2 (52). In the *in vivo* MCAO model and *in vitro* Neuron 2A cell line (transfected with PPARγ-specific) OGD reperfusion model, PPARγ-deficient cells displayed an extended infarct trigon and more severe neuron deficiency, and upregulated levels of CHOP, as well as cleaved caspase-12, and binding immunoglobulin protein (BiP) (both markers of ER stress). It has been proven that PPARγ protects the brain from CI/RI by suppressing ER stress (53).

In summary, PPARγ reduces the production of ROS by inhibiting the activity of Rac1 and NOX on the one hand and increasing the expression of antioxidant enzymes on the other hand, thereby reducing the cell damage caused by MDA and LDH. In addition, PPARγ can also against the brain from CI/RI injury by inhibiting ER stress.

## Anti-Inflammatory Effects of PPARγ in CI/RI

**Figure 1:**
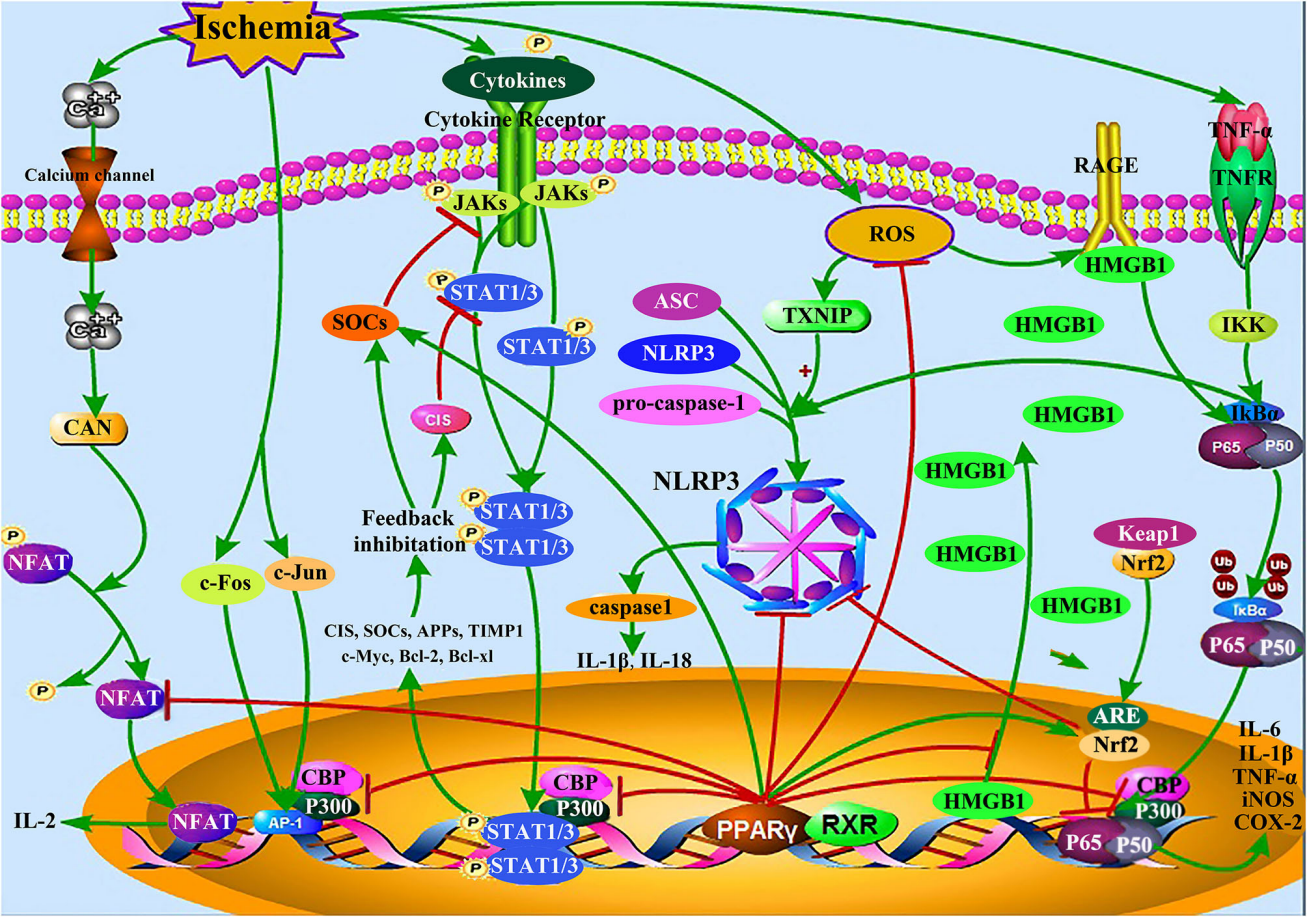
Inflammation-related signaling pathways in CI/RI mediated by PPARγ. PPARγ competitively inhibits JAK-STAT, NF-κB, AP-1, NFAT, HMGB1/RAGE, and other inflammation-related signaling pathways, up-regulates the expression of Nrf2, and SOCS protein, reduces the expression of NLRP3 inflammasome and pro-inflammatory mediator including IL-1β, IL-6, and TNF-α, thereby reducing the CI/RI injury caused by inflammation.

Inflammation, a critical process in CI/RI (54); it is mediated by a range of endogenous factors, including inflammatory cytokines [IL-1, IL-6, TNF-α, interferon gamma (IFN-γ), etc.], lipid mediators, and eicosanoids, including prostaglandins, leukotrienes, and lipoxin (55). Inflammatory cascade reactions gradually induce apoptosis in the peri-ischemic region of the brain (54). PPARγ agonists can inhibit inflammation induced by CI/RI by competitively inhibiting the production of inflammatory factors and their signaling pathways (56); more specifically, by modulating the JAK-STAT proteins, NF-κB, nuclear factor of activated T-cells (NFAT), and activator protein 1 (AP-1) pathways, which regulate the expression of nuclear factor erythroid 2-related factor 2 (Nrf2), nucleotide oligomerization domain (NOD)-like receptor protein 3 (NLRP3), and high mobility group box protein 1 (HMGB1) (Figure 1).

### PPARγ Inhibits the JAK-STAT Pathway and Increases Suppressor of Cytokine Signaling (SOCS) Protein Expression

It has been shown that CI/RI can induce the release of a large number of inflammatory cytokines, such as IL-1, IL-6, TNF-α, and IFN-γ. These factors can activate the JAK-STAT signaling pathway and produce inflammation (57). The specific mechanism responsible for this process is that a variety of extracellular inflammatory cytokines bind to the corresponding receptors on the membrane as ligands, causing the receptor-coupled JAK to aggregate and be activated by phosphorylation, resulting in the subsequent phosphorylation and activation of transcriptional activation factor STAT (44). Activated STAT leaves the receptor and dimerizes to form a homodimer/heterodimer that translocates into the nucleus to bind to the coactivator cyclic adenosine monophosphate response element-binding protein (CREB)-binding protein (CBP) or p300. There, it can then bind to the promoter of the target gene, thereby activating its expression. The STAT family has seven members that mediate the transcription of multiple cytokines: Stat1 and Stat2 are required for IFN-γ to regulate gene expression; Stat3 is required for gene expression in IL-6-induced acute phase responses; STAT1 and STAT3 can reduce the expression of anti-apoptotic genes [Bcl-2 and B-cell lymphoma extra-large (Bcl-xl)], leading to neuronal apoptosis. As a target gene of STATs, the SOCS protein can inhibit their activation directly or inhibit the activation of JAKs, specifically, by binding to JAKs or associated receptors, thus playing a regulatory role through negative feedback (44, 58).

A study reported that activated PPARγ forms an isomeric dimer with the retinoid X receptor (RXR), known as PPARγ-RXR. This dimer binds CBP and p300 through competition and recruitment, thereby inhibiting STAT1 activation and blocking the production of STAT1-related proinflammatory factors (IL-6, IL-1, and TNF-α) (59). PPARγ agonists can inhibit the phosphorylation of JAK1/2 and STAT1/3 in the reperfusion zone of ischemia-reperfusion (IR)-injured rats, decrease their neurological score, and reduce the IR injury in brain tissue (5, 58). In addition, PPARγ inhibits the JAK-STAT pathway by promoting the expression of SOCS, and studies have shown that the PPARγ agonists 15d-PGJ2 and rosiglitazone are effective inducers of SOCS. Treatment with rosiglitazone can induce the expression of SOCS3 and prevent phosphorylation of JAK2 and STAT3 2 h after focal IR, thereby playing a neuroprotective role (49, 60).

### PPARγ Inhibits the NF-κB Pathway

CI/RI can also initiate activation of the NF-κB pathway. Normally, NF-κB binds to its inhibitors (IκBα and IκBβ) to form a trimer, which is distributed throughout the cytoplasm in an inactive state (59). In inflammation induced by CI/RI, the TNF receptor-associated factor (TRAF) protein family induces the activation of inhibitor of NF-κB (IκB) kinase (IKK), leading to the phosphorylation of IκB proteins and the subsequent dissociation of IκB from the heterotrimeric p50/p65/IkB complex, which is degraded by the proteasome after ubiquitination, ultimately resulting in the activation of NF-κB. Free NF-κB then enters the nucleus and binds to p300/CBP, followed by specific binding to certain kB sites on DNA to induce transcription and expression of related genes, including proinflammatory cytokines such as IL-1β, IL-6, and TNF-α, and neuroinflammatory genes such as COX-2 and iNOS (56, 59, 61). Among these, COX-2 is an enzyme that aggravates the changes induced by CI/RI and promotes the conversion of arachidonic acid to prostaglandin H2 (PGH2), which produces prostaglandin E2 (PGE2), and participates in the inflammatory response (60).

Several *in vivo* and *in vitro* studies have shown that PPARγ reduces the release of proinflammatory cytokines in CI/RI models by inhibiting the NF-κB pathway (56). PPARγ binds to the P65/P50 subunit of NF-κB directly to form a transcriptional inhibitory complex that reduces the binding activity of NF-κB at DNA sites, thereby inhibiting the production of downstream inflammatory factors. PPARγ also inhibits NF-κB transcription by competitively binding the coactivator p300 and CBP (59). The PPARγ agonist 15dPGJ2 mediates its anti-inflammatory effects by inducing binding of PPARγ to IKK directly, thereby inactivating it (62). As a novel PPARα/γ dual agonist, N15 can enhance PPARα/γ signal transduction and inhibit the activation of the NF-κB signaling pathway, thus playing an anti-inflammatory role that relieves brain damage after CI/RI (19).

### PPARγ Regulates the Expression of Nrf2

Nrf2 is an important transcription factor that mediates oxidative stress. Physiologically, Nrf2 binds to the cytoplasmic adaptor protein Kelch-like ECH-associated protein 1 (Keap1) to inhibit its activity. In ischemia-induced oxidative stress, Nrf2 uncouples from Keap1 and translocates into the nucleus where it interacts with the anti-oxidative response element (ARE) to induce the expression of downstream protective phase II detoxifying enzymes and antioxidant enzymes to achieve cytoprotective effects. There is increasing evidence that activation of the Nrf2 pathway significantly inhibits NF-κB and that it plays a protective role in the inflammatory response (63). Nrf2 and PPARγ genes are mutually regulated. First, the Nrf2 gene sequence contains the PPRE, while the PPARγ gene sequence contains the Nrf2-specific response element (i.e., ARE). Second, PPARγ and Nrf2 share some common target genes. For example, the DNA sequence of CAT contains both ARE and PPRE, which can be regulated by Nrf2 and PPARγ at the same time to enhance the expression of CAT. Third, both PPARγ and Nrf2 regulate NF-κB negatively, thus inhibiting NF-κB synergistically (64). Furthermore, in an *in vitro* CI/RI model, microglial Nrf2 plays a negative role in the activation of the NLRP3 inflammasome (65).

Huang et al. (66) found that ginsenoside G-Rg1, an effective activator of PPARγ, downregulated Nrf2 levels in the cytoplasm and upregulated Nrf2 levels in the nucleus. A study by Hsu et al. (67) found that monascin, a novel PPARγ agonist, could also activate Nrf2, and Li et al. (56) showed that luteoloside attenuated neuroinflammation in a focal cerebral ischemia model in rats by modulating the PPARγ/Nrf2/NF-κB signaling pathway.

### PPARγ Downregulates the Expression of NLRP3

The NLRP3 inflammasome is a multiprotein complex composed of NLRP3, apoptosis-associated speck-like protein containing a C-terminal caspase recruitment domain (ASC), and procaspase-1. Recently, it was shown that thioredoxin-interacting protein (TXNIP) activation is a key event linking oxidative stress to inflammation and apoptosis in neurons (68). Mitochondria respond to oxidative stress induced by IR and ROS, prompting TXNIP to dissociate from the complex and rapidly bind to NLRP3 inflammasomes to induce their activation (69); this subsequently triggers the activation of caspase-1 and the cleavage of pro-IL-1β and pro-IL-18, thereby inducing cell death and the release of many intracellular pro-inflammatory molecules (70). In addition, NF-κB and mitogen-activated protein kinase (MAPK) signaling promotes NLRP3 inflammasome activation in neurons following ischemic stroke (71). Hong et al. (72) showed that MCC950, the specific inhibitor of NLRP3 reduced the neurological deficit score and improved the 28-day survival rate of CI/RI in diabetic mice.

In a recent study, PPARγ binding sites were found in the promoter regions of a member of the NLRP3 family, indicating a correlation between PPARγ activity and the NLRP3 family of proteins (69). It is reported that PPARγ is a negative regulator of NLRP3 inflammasome activation (73); for example, umbelliferone (UMB) may partially inhibit the activation of the TXNIP/NLRP3 inflammasome by activating PPARγ, thereby reducing the levels of IL-1β, and IL-18 in brain tissue and reducing brain damage caused by focal ischemia (69). Similarly, the PPARγ agonist pioglitazone has been shown to ameliorate retinal ischemia/reperfusion injury by suppressing NLRP3 inflammasome activities (74).

### PPARγ Downregulates the Expression of HMGB1/RAGE

HMGB1, a non-histone DNA-binding protein, has been identified as an important late pro-inflammatory mediator whose effect depends on the translocation of HMGB1 from the nucleus to the cytoplasm and its subsequent release into the extracellular space (75). HMGB1 localization is dependent upon the acetylation of lysine residues in its two nuclear localization signals (NLS1/2), and most non-acetylated HMGB1 is stored in the nucleus (76). Under CI/RI stimulation, hyperacetylation of the NLS sites on HMGB1 leads to the reduced affinity of HMGB1 for DNA, so HMGB1 is passively excreted by damaged and necrotic cells or is actively secreted by activated immune cells, leading to further immunological amplification (11, 76).

The receptor for advanced glycation end product (RAGE) is a transmembrane innate immune receptor, and the activation of this receptor plays an important role in mediating pro-inflammatory effects. Once HMGB1 is released from the cell into the extracellular space, it can bind to RAGE at the cell membrane, resulting in the activation of MAPK and NF-κB/p53 signaling pathways, which mediate the production of various downstream pro-inflammatory cytokines (11).

Le et al. (77) first found that glycyrrhizin (GLY), the specific inhibitor of HMGB1, reversed the hypoxia-ischemia insult-induced loss of neurons and myelin in the hippocampal region and neurobehavioral impairments, which was achieved through the inhibition of HMGB1 expression and nucleocytoplasmic translocation, the inflammatory response, the suppression of increases in microglia/astrocytes, and the inhibition of hippocampal cell apoptosis.

PPARγ is capable of suppressing HMGB1 acetylation; this reduces the secretion of HMGB1, thereby reducing brain damage caused by late inflammation in CI/RI (78). In models of OGD models of neuronal cells *in vitro*, a study showed that rosiglitazone downregulated the expression of RAGE by upregulating PPARγ, and the secretion of HMGB1 decreased significantly, which could be reversed by administration of the PPARγ receptor inhibitor GW9662 (79). Pioglitazone has also been shown to mediate the downregulation of cytoplasmic translocation of HMGB1 and RAGE after IR, suggesting that this PPARγ-dependent pathway may be involved in pioglitazone's inhibition of HMGB1/RAGE activation (11).

### PPARγ Inhibits the NFAT Pathway

NFAT is a family of transcription factors that is mainly expressed in immune cells and exists in the cytoplasm in an inactive phosphorylation state (59). Calcineurin (CaN) is the serine/threonine protein phosphatase that is only regulated by calmodulin (CaM), which mainly catalyzes the dephosphorylation of phosphatidylserine and phosphatidylthreonine, and the main substrates, *in vivo*, are the NFAT family proteins (80).

CI/RI causes activation of NMDA receptors and other Ca^2+^ channels, as well as intracellular Ca^2+^ overload. Activated Ca^2+^-dependent CaN binds to the highly conserved sequence region of NFAT, leading to its dephosphorylation; following exposure to nuclear localization signals, it undergoes nuclear translocation, increasing the activity of the downstream pro-inflammatory factor IL-2 (59, 80).

Yang et al. (81) found that in T-cell-mediated inflammation, PPARγ exerts an anti-inflammatory effect by binding to ligands that can inhibit the expression of IL-2, either by inhibiting NFAT binding to its DNA target region and the subsequent transcription, or by inhibiting interactions between certain proteins. In addition, Raman et al. (82) showed that the PPARγ agonist 15d-PGJ2 reduced the translocation of NFAT into the nucleus, decreasing its activity, resulting in lower IL-2 expression; the addition of a PPARγ antagonist partially reversed these effect of 15d-PGJ2 on NFAT transcription.

### PPARγ Inhibits the AP-1 Pathway

AP-1 is an important nuclear transcription factor, as different combinations of Jun (c-Jun, junB, junD) and Fos [c-fos, fosB, fos-related antigen-1(fra-1)] family proteins determine the composition of active hetero/homodimers inside the cell and the activity of the genes that they regulate (59). The stimulation of external signals such as those induced by CI/RI can activate the transcription of immediate early genes c-Fos and c-Jun, and the later translation into nuclear proteins, such as those of the Fos and Jun families. Fos and Jun family proteins combine to form AP-1, which is a transcriptional regulatory protein. After re-translocating into the nucleus, AP-1 binds to the DNA regulatory region of the target gene, thus regulating its transcriptional efficiency and expression, in addition to playing a messenger role in the signaling cascades (83).

In inflammatory reactions, AP-1 can induce apoptosis and the synthesis of adhesion and inflammatory factors. Studies have shown that PPARγ inhibits the AP-1 signaling pathway to reduce the expression of inflammatory cytokines by competing with AP-1 for binding to the coactivators p300 and CBP; thus it plays a neuroprotective role by inhibiting the infiltration and cytotoxic effects of inflammatory cells in CI/RI (59, 81). Oleic acid is an endogenous agonist of PPARγ, which can downregulate the expression of inflammatory factors in CI/RI, possibly due to the antagonistic effect of PPARγ on AP-1 signal transduction, which can be abolished by the PPARγ antagonist GW9662 (20).

In summary, PPARγ reduce CI/RI injury by inhibiting multiple inflammatory pathways and inflammatory cytokines, thus playing a neuroprotective role.

## The Role of PPARγ in Inhibiting Microglial Activation and Regulating their Phenotype During CI/RI

Microglia are mononuclear phagocytes located in the CNS that, at physiological rest, monitor the microenvironment and clear apoptotic tissue. In the ischemic lesions induced by the MCAO model, microglia are rapidly activated and recruited from the peripheral region to the lesion site, and the number of microglia in the ischemic core increases and the cell body size and total branch length decrease (84). The change from “branch-like” to “shrub-like” and then to “amoeba-like,” presenting a CD11b+ branched amoeba-like morphology (84). Microglia may also polarize into multiple phenotypes. It is currently believed that microglia have at least two polarized states: type M1 and type M2 (85). M1-type activated microglia secrete pro-inflammatory cytokines, such as TNF-α and IL-1β, contributing to the inflammatory response and leading to worse outcomes after cerebral ischemia. M2-type activated microglia generate IL-10 and IL-4, transforming growth factor-β (TGF-β), and certain cell repair factors, all of which drive the resolution of neuroinflammation and anti-inflammatory processes (86).

After CI/RI, microglia are activated by a variety of pro-inflammatory factors, chemokines, NO, and ROS, among others, and are converted into the M1 and M2 phenotypes, which try to maintain a balance between anti-inflammatory and pro-inflammatory states. However, M2-type microglia only exert anti-inflammatory effects in the early stage of ischemia, and gradually shift from M2-type to M1-type microglia in the middle stage, as microglia begin to shift from anti-inflammatory to pro-inflammatory responses (87). Moreover, some studies have shown that the number of activated microglia was positively correlated with the degree of CI/RI, and activated microglia trigger local inflammatory infiltration and injury of brain tissue (88). Therefore, inhibition of microglial activation and polarization is considered an effective strategy for the treatment of IR.

A large number of studies have shown that agonist-induced activation of PPARγ not only inhibits microglial activation, but also participates in the regulation of microglial phenotypes. Ionized calcium binding adaptor molecule 1 (Iba1) is a microglia/macrophage-specific protein antibody; relative to vehicle-treated MCAO model mice, those treated with the PPARα/γ dual-agonist aleglitazar displayed a significant reduction in Iba1+ cells in the ischemic MCA territory, suggesting that aleglitazar reduces microglial activation (89). In adult rodents, pretreatment with rosiglitazone or pioglitazone 1 day prior to the induction of ischemia resulted in decreased microglial activation and macrophage infiltration, as well as decreased expression of pro-inflammatory mediators COX-2, iNOS, and IL-1β mRNA in the ischemic hemisphere (90). Recent studies indicate that PPARγ agonists attenuate ischemia-induced activation of microglia, expression of intracellular adhesion molecule 1 (ICAM-1), and neutrophil infiltration in C57BL/6 mice (91). The present results showed that toll-like receptor 4 (TLR4) was localized to CD11b/c-positive cells (microglia/macrophages) after CI/RI, and N15 treatment markedly reduced microglia/macrophage activation and TLR4 expression (19).

Studies have shown that XQ-1H, a novel derivative of ginkgolide B, increased the expression of PPARγ in OGD reperfusion injury BV-2 cells (microglia); incubation with XQ-1H protected BV2 cells from OGD reperfusion injury, decreased the co-expression of CD16 (a marker for M1-type microglia) and Iba1, and increased the co-expression of CD206 (a marker for M2-type microglia) and Iba1. All these effects could be inhibited by administration of the PPARγ inhibitor GW9662. The results proved that XQ-1H regulated the polarization of microglia by promoting the anti-inflammatory phenotype and inhibiting the pro-inflammatory phenotype (21). White matter is composed mainly of axonal fibers, oligodendrocytes, and other glial cells, and is highly vulnerable to ischemic injury. White matter injury contributes to nearly half of the infarct volume seen following human ischemic stroke (30). It has been confirmed that rosiglitazone treatment improved white matter integrity in an *in vivo* CI/RI model, at least in part by promoting oligodendrogenesis and facilitating microglial polarization toward the beneficial, anti-inflammatory M2 phenotype (30). Furthermore, when M2 microglia were depleted from the mixed glial culture system, rosiglitazone was less effective in inducing oligodendrogenesis (30).

In conclusion, PPARγ can both inhibit microglial activation and promote microglial polarization toward a favorable M2 phenotype, thereby reducing inflammatory responses caused by microglial activation, and exert neuroprotective effects.

## The Role of PPARγ in Maintaining the BBB During CI/RI

Neurovascular unit (NVU) is regarded as the basic structural and functional unit of brain, and BBB is a vital component of the neurovascular unit which is composed of tightly connected basement membranes of capillary endothelial cells, pericytes, and astrocyte endpoints (92). Numerous studies have confirmed that the important pathological processes occurring in the early stage of ischemia/reperfusion are accompanied by changes in BBB structure and function, which involved different factors including matrix metalloproteinases (MMPs), aquaporin (AQP), claudin, occludin, and inflammatory modulators (93). Pathophysiological changes occur in endothelial cells in the early stage of ischemia/reperfusion, which activate the in-born immune system and cause paracellular hyperpermeability in endothelial cells, and then recruitment of leucocytes causes endothelial barrier failure and activation of MMP (94, 95). Activated MMPs cause disruption of TJs, which lead to increased paracellular permeability (96). Alongside, the stressed components of the NVU release a myriad of chemokines and cytokines (97). Activated microglia and an increased level of inflammatory molecules (IL-1β and TNF-α) and ROS increase the number of adhesion receptors on endothelial cells permitting infiltration of leukocytesand other molecules which add to the BBB permeability precipitating vasogenic edema. These events ultimately can culminate into hemorrhagic transformation (HT) (96). Notably, the breakdown of BBB leads to brain influx of blood-derived vasculotoxic and neurotoxic macromolecules, causing reductions in capillary blood flow due to microvascular degeneration and pericapillary edema, depriving metabolically active neurons of oxygen, and other essential nutrients on the one hand and accumulating neurotoxins on the other hand, initiating neuronal functional and structural changes ultimately (98).

MMPs are a group of Zn-containing proteases that can degrade all the components that make up the extracellular matrix (99). MMP-9 is an important member of the MMP family; it can degrade claudin-5 and other BBB components, and it participates in the pathological mechanisms involved in the destruction of the BBB, changing the vascular permeability and leading to the formation of vasogenic cerebral edema (100). Leukocytes, especially neutrophils, are the main source of MMP-9 following brain ischemia (101). In CI/RI, NF-κB, and AP-1, which are involved in pro-inflammatory processes, bind to the transcriptional regulatory domain of MMP-9, promoting its gene transcription. In addition, in these processes, MMP-2, and MMP-9 are activated and tissue inhibitor of metalloproteinase (TIMP-1, an MMP-9-specific inhibitor) is inhibited, resulting in an imbalance of pro-MMP/anti-MMP-related mechanisms; in contrast, a 30% reduction in infarct volume is observed following intravenous injection of TIMP-1 (99, 102). In addition, mifepristone, which acts as a PPARγ agonist, has been shown to attenuate CI/RI by restoring the balance between MMPs and TIMPs and inhibiting inflammatory cytokines to maintain BBB homeostasis (103).

AQP4 is one of the most abundant molecules in the brain, and is a predominantly-expressed water channel molecule in astrocytic membranes at the BBB and brain-liquor interfaces. Beta-caryophyllene inhibits the expression of AQP4 and increases the expression of claudin-5 and the occludin enzyme by activating PPARγ, thereby reducing BBB damage and edema in CI/RI (93).

As a proinflammatory cytokine, NLRP3 contributed to the regulation of MMP2/-9 and tight-junction protein expressions and endothelial cell permeability (104). Yang et al. (104) further showed that the contribution of NLRP3 to neurovascular damage was associated with an autocrine/paracrine pattern of NLRP3-mediated IL-1βrelease. Wang et al. (105) found that MCC950, the inhibitor of NLRP3, suppressed the expression of AQP4 and endothelin-1(ET-1) to reduce the accumulation of water and alleviate cerebral edema, suggesting that NLRP3 may be a potential molecular target for reducing neurovascular damage.

HMGB1 can trigger downstream inflammatory responses in CI/RI model. In the process, HMGB1 triggers three specific downstream receptors, RAGE, TLR-2 and TLR-4 (106), which further induce the expression of NF-κB, MMP, and MAPK (107, 108). The inflammatory cascade is involved in BBB disruption, apoptosis and nerve damage, and further aggravating brain damage. Anti-HMGB1 antibody can inhibit the morphological and functional changes in the BBB induced by HMGB1 (106). Telmisartan significantly decreased the number of Iba1-positive cells expressing HMGB1 and decreased plasma HMGB1 levels in PPARγ-dependent manner, thus improving BBB damage (109).

In addition to affecting the above key cytokines and proteases, PPARγ also protects the BBB by inhibiting the Ras homolog A (RhoA)/Rho-associated kinase (ROCK) signaling pathway. RhoA is a member of the Ras superfamily of GTP-coupled proteins, and ROCK is a downstream serine/threonine kinase of RhoA. Cerebral ischemia activates tyrosine kinase (TK) and G protein-coupled receptors (GPCRs). Activated TK and GPCRs further activate RhoA, which then acts on the Rho-binding domain in the ROCK intermediate coiled-coil region to remove the inhibition maintained by the carboxyl-terminal PH structural region, ultimately activating ROCK (110). Activated ROCK causes damage to the BBB by phosphorylating the myosin light chain (MLC), which opens up the intercellular tight junction and increases endothelial intercellular permeability. Thus, the RhoA/ROCK signaling pathway exerts a regulatory effect on vascular endothelial function, and BBB permeability in CI/RI can be regulated by intervening in this pathway's altered signaling (110, 111). Studies have shown that after MCAO, PPARγ activated by pioglitazone resulted in decreased activity of RhoA and ROCK, decreased expression of MMP-9 and MDA, increased expression of the vascular endothelial tight junctions, occludin, SOD, and GSH-Px, and, finally, decreased infarct volume and maintenance of BBB integrity in rat brain tissue (110).

In short, NVU is a structural and functional whole. The multifactorial pathophysiology of CI/RI involving several components such as neurons, astrocytes, and endothelial cells which continually interact with each other has made CI/RI research very difficult. A lot of clinical and laboratory data show that protect the BBB after cerebral ischemia should be a priority for preclinical ischemic stroke investigations. And PPARγ can play a role in multiple regulation of BBB, thereby playing a protective role.

## The Role of PPARγ in Promoting Angiogenesis and Neurogenesis During CI/RI

### Protective and Regenerative Effects of PPARγ on Blood Vessels

Damage to cerebrovascular endothelial cells, the main structural components of the BBB, results in the production of a variety of pro-inflammatory factors in the ischemic brain, which further exacerbate ischemic brain damage (112). Baculoviral inhibitor of apoptosis (IAP) repeat-containing 5 (BIRC5; also known as survivin) belongs to the IAP gene family and has recently been found to play a protective role in cerebrovascular endothelial cell injury. OGD experiments in mouse cerebral microvascular endothelial cells (bEnd.3) have shown that OGD-treated cells transfected with the PPARγ overexpression plasmid stimulated the proliferation of bEnd.3 cells and reduced their apoptosis. In addition, results from electrophoretic mobility shift/supershift and chromatin immunoprecipitation assays suggested that PPARγ can bind to the promoter of BIRC5, and PPARγ can increase the expression of BIRC5 at the mRNA and protein levels, proving that PPARγ may protect the cerebral microvascular endothelium against IR injury by enhancing the activity of BIRC5 (113). VEGF is a highly specific vascular endothelial cell growth factor that can promote increased vascular permeability, the denaturation of extracellular matrix proteins, the migration and proliferation of vascular endothelial cells, and the formation of blood vessels. PPARγ activation also increases VEGF expression in vascular smooth muscle cells (114). PPARγ coactivator 1α (PGC-1α), a transcriptional coactivator, is a known regulator of VEGF gene transcription (115). It is reported that a significant upregulation of KDR (VEGF receptor-2) in the ischemic hemisphere of mice is induced by treatment with the PPARα/γ dual agonist aleglitazar, which promotes angiogenesis after MCAO (89).

### Effects of PPARγ on Neurogenesis and Cell Differentiation

There have been descriptions of PPARγ's involvement in pathways that are also involved in the control of the proliferation, migration, and differentiation of neural stem cells (116). The subventricular zone (SVZ), located in the lateral wall lining the lateral ventricle, harbors the largest population of neural stem cells capable of generating new neurons, astrocytes, and oligodendrocytes (12). In the ischemic model, the agonist pioglitazone activates PPARγ, which augments the proliferation of resident stem cells in the SVZ and the recruitment of mesenchymal stem cells in bone tissue; PPARγ is involved in the enhanced migration of both types of stem cells from the SVZ to the peri-infarct area, where they differentiate into mature neurons, glia, and blood vessels in association with activated protein kinase B (Akt), microtubule-associated protein 2 (MAP2), and VEGF (12). Oligodendrocytes are mainly involved in the formation of myelin sheaths and are highly sensitive to ischemic injury. In a CI/RI model, injured mature oligodendrocytes no longer produce functional myelin; since mature oligodendrocytes are not proliferative, successful regeneration of oligodendrocytes is essential for remyelination after brain injury. According to reports, rosiglitazone not only enhanced the proliferation of OPCs, but also promoted OPC differentiation into mature myelinating oligodendrocytes (30).

In addition, neurotrophic factors regulate the survival, proliferation, and differentiation of cells in the CNS (12, 117). PEDF has been established as a neuroprotective factor that promotes the survival of various types of neurons by increasing their resistance to neurotoxic damage, especially that induced by ischemic stroke (118). Experiments have shown that PPARγ mediates the function of PEDF in regulating survival, proliferation, and differentiation of cells in the CNS (118). In the MCAO rat model, the PPARγ agonist G-Rg1 significantly upregulates the expression of TGF-β1 and brain-derived neurotrophic factor (BDNF) in the hippocampal CA1 region, increases the expression of VEGF, nerve growth factor, and Bcl-2, and enhances the formation of new synapses (4).

In summary, PPARγ promotes proliferation and differentiation of neural stem cells and proliferation of vascular endothelial cells, increases expression of neurotrophic factors and VEGF, and thus promotes neurogenesis and angiogenesis.

## Anti-Apoptotic Effect of PPARγ in Cerebral CI/RI

In CI/RI, PPARγ plays a complex multi-mechanistic neuroprotective role involving modulation of many processes, including inhibiting inflammation, reducing the level of oxidative stress, reducing the production of pro-apoptotic factors, and promoting the expression of anti-apoptotic factors. After IR, the production of ROS increases, leading to damage to intracellular biofilm lipids (for example, MDA), proteins and nucleic acids, mitochondrial damage, and the evoked release of apoptosis inducing factor (AIF) and cytochrome C (Cyt-C) in mitochondria (4). These upregulated levels of AIF and Cyt-C induce the activation of a downstream apoptotic cascade that includes the activation of cleaved caspase-3 and cleaved caspase-9, both of which regulate the levels of anti-apoptotic proteins (4, 119, 120). The activation of PPARγ during ischemic injury can dependently inhibit the NF-κB signaling pathway, known as the PPARγ-ERK-NF-κB signaling pathway, to reduce the secretion of iNOS, gelatinase B, and scavenger receptor A, thereby inhibiting the expression of the pro-apoptotic protein caspase-3 and promoting the expression of the anti-apoptotic protein Bcl-2, which can play a protective role in the nervous system (121). PPARγ reduces the synthesis of IFN-γ and iNOS by inhibiting the JAK-STAT pathway, thereby regulating apoptosis (121). PPARγ can also prevent IR-induced neuronal apoptosis by upregulating the response element heme oxygenase-1 (HO-1), which has cytoprotective properties, and its anti-apoptotic effects may be based, in part, on its inhibition of inflammatory factors and its antioxidative function (122).

In *in vivo* MCAO and OGD models, the PPARγ agonist pioglitazone could effectively antagonize the neuronal pyroptosis caused by ischemia and hypoxia by inhibiting pyroptosis-related proteins such as caspase-1, the NLRP3 inflammasome, and ASC, and by reducing the release of cytokines such as IL-1β and IL-18 (11). Ou et al. detected PPARγ-positive cells on the ischemic side of the middle cerebral artery and found that treatment with the natural agonist 15d-PGJ2 reduced infarct size, the expression of caspase-3, the necrotic cascade response, and apoptosis (6, 123). In addition, dual-specificity phosphatase (DUSPs) acts specifically on JNK and p38, and studies have shown that the PPARγ agonist rosiglitazone induces phosphorylation of p38 and JNK MAPK in neurons, and inhibits neuronal apoptosis in an animal model of cerebral ischemia, which was mainly achieved by promoting the upregulation of DUSP8 and Bcl-xl (79).

Poly ADP-ribose polymerase-1 (PARP-1) is a protease ubiquitously expressed in eukaryotic cells that plays a key role in sensing and regulating cellular stress and repairing damage. Activation of PARP-1 promotes DNA repair and maintains genomic stability when DNA damage occurs, and the failure to repair DNA damage may trigger apoptosis and activation of caspase signaling. In the above process, PARP-1, as a substrate of caspases, is considered to be one of the markers of apoptotic initiation. Following excessive PARP-1 activation induced by the free radicals that are produced during cerebral IR, AIF, as a promoter of cell death, is released into the cytoplasm and then enters the nucleus to induce chromatin aggregation and DNA fragmentation, ultimately inducing PART-1-dependent cell death (124). Therefore, inhibition of PARP-1 activation has a neuroprotective effect. Jui-Sheng Wu and other studies have shown that in the OGD model, the analysis of the proapoptotic markers found greatly enhanced levels of caspase-3 and PARP-1 proteins, which were relieved by PPARγ agonist 15d-PGJ2, which could be reversed by administration of the PPAR receptor inhibitor GW9662 (41).

In summary, PPARγ reduces the Parthanatos process and the apoptotic cascade by promoting DUSP8 upregulation, inhibiting the PARP-1 overactivation and the expression of caspase-3, and thereby plays a neuroprotective role in CI/RI.

## Discussion

CI/RI is a complex pathological process. The damage and cascade of reactions caused by CI/RI are related to oxidative stress, inflammation, cytokine-mediated damage, glutamate excitotoxicity, intracellular calcium overload, and many other factors. These factors interact with each other, ultimately leading to apoptosis or neuronal necrosis in the ischemic region. However, there is currently no effective treatment for ischemic brain injury. So far, intensive study of PPARγ has concentrated on its regulatory role in inflammation, atherosclerosis, insulin resistance, glucose metabolism, obesity, and tumor formation.

CI/RI markedly induced PPARγ nuclear translocation and the up-regulation of protein level, which relocalization suggested the activation of PPARγ (125). Xu et al. (126) found that after 60 min of ischemia, a time-dependent enhancement in PPARγ translocation was demonstrated by the analysis of cytosol and nuclear level of PPARγ at 2, 4, 8, and 24 h of reperfusion. It was worthy of note that this nuclear translocation of PPARγ occurred as early as 2 h of reperfusion, at which time point no detectable increase in total PPARγ expression was observed, implying that the onset of translocation was earlier than that of the up-regulation of protein expression, indicating that nuclear translocation of PPARγ was a quick response to I/R (126).

At present, the mechanism of CI/RI is still not completely understood, and the PPARγ pathway is intricate. Our review shows that PPARγ plays a potentially protective role against CI/RI through its interaction with multiple pathways and its multi-targeted effects. Many studies have shown that PPARγ agonistic ligands such as 15d-PGJ2, pioglitazone, troglitazone, and rosiglitazone exerted their neuroprotective effects by promoting the activation and expression of PPARγ, and by enhancing its anti-inflammatory effects, reducing the levels of oxidative stress and ER stress, increasing the expression of antioxidants, maintaining BBB integrity, inhibiting the activation of microglia and regulating their phenotype, and promoting nerve regeneration, angiogenesis, and reducing apoptosis. However, animal and cell models are often used in the related study of the neuroprotective effect of PPARγ in CI/RI, and there is a lack of large-scale clinical research at present. Therefore, with the development of molecular biology, bioinformatics, and other related subjects, we can more thoroughly and precisely explore the activation state and the independent mechanisms of PPARγ to develop more effective PPARγ agonists, and to find the most effective target for the treatment of CI/RI injury. Ultimately, the study of PPARγ is expected to provide a new therapeutic approach for the treatment of ischemic cerebrovascular diseases, and it is important for the prevention of neurodegenerative diseases such as Parkinson's disease, Alzheimer's disease, and Huntington's disease.

## Author Contributions

JK, YD, and BS devised the review. JK and YD conducted literature review and provided the first draft. JK, SL, and YX created the figures. All authors contributed to manuscript revision and approved the submitted version.

## Conflict of Interest

The authors declare that the research was conducted in the absence of any commercial or financial relationships that could be construed as a potential conflict of interest.
